# Adverse childhood experiences and crime outcomes in early adulthood: A multi-method approach in a Brazilian birth cohort

**DOI:** 10.1016/j.psychres.2024.115809

**Published:** 2024-04

**Authors:** Andreas Bauer, Rafaela Costa Martins, Gemma Hammerton, Maurício Scopel Hoffmann, Andressa Souza Cardoso, Camila Colvara, Clarissa Fialho Hartmann, Gabriel Calegaro, Luciana Rodrigues Perrone, Nilvia Aurélio, Ana M.B. Menezes, Joseph Murray

**Affiliations:** a Postgraduate Program in Epidemiology, Federal University of Pelotas, Pelotas, Brazil; b Human Development and Violence Research Centre (DOVE), Federal University of Pelotas, Pelotas, Brazil; c Centre for Academic Mental Health, Population Health Sciences, Bristol Medical School, University of Bristol, Bristol, UK; d Medical Research Council Integrative Epidemiology Unit at the University of Bristol, Population Health Sciences, Bristol Medical School, University of Bristol, Bristol, UK; e Department of Neuropsychiatry, Federal University of Santa Maria, Santa Maria, Brazil; f Mental Health Epidemiology Group (MHEG), Universidade Federal de Santa Maria, Santa Maria, Brazil; g Graduate Program in Psychiatry and Behavioral Sciences, Universidade Federal do Rio Grande do Sul, Porto Alegre, Brazil

**Keywords:** Adverse childhood experiences, Crime, Cumulative risk, Latent class analysis, Network analysis

## Abstract

This study aimed to investigate alternative approaches to a cumulative risk score in the relationship between adverse childhood experiences (ACEs) and crime. Using data from the 1993 Pelotas (Brazil) Birth Cohort (*n* = 3236), we measured 12 ACEs up to 15 years, and past-year violent and non-violent crime at 22 years. We used four analytical approaches: single adversities, cumulative risk, latent class analysis, and network analysis. When examined individually, physical abuse, emotional abuse, and domestic violence were associated with both crime outcomes, whereas maternal mental illness and discrimination were associated with violent crime only, and parental divorce and poverty with non-violent crime only. There was a cumulative effect of ACEs on crime. The class with child maltreatment and household challenges was associated with both crime outcomes; exposure to household challenges and social risks was associated with violent crime only. In network models, crime showed conditional associations with physical abuse, maternal mental illness, and parental divorce. Although cumulative ACEs did associate with crime, some individual and combinations of ACEs showed particularly strong and robust effects, which were not captured by the cumulative score. Many ACEs are closely connected and/or cluster together, and the usefulness of the ACE score needs to be further evaluated.

## Highlights

• A cumulative score may hide important differences in risk for crime outcomes.
• Cumulative adverse childhood experiences were associated with crime.
• ACEs – individually, cumulatively, and as clusters – were associated with crime.
• Some individual and/or combinations of ACEs showed particularly strong effects.
• Some ACEs were more strongly linked to each other than others.

## 1 Introduction

The concept of adverse childhood experiences (ACEs) was first introduced in a classic study by Felitti et al., using data from US adults with a Kaiser Permanente health plan (Felitti et al., 1998). Retrospective reports on *child abuse* (physical, emotional, and sexual) and *household dysfunction* (domestic violence, substance abuse, mental illness, and incarceration) were associated with some of the leading causes of death (Felitti et al., 1998). This and many subsequent studies, which have also examined other ACEs, such as parental divorce and *child neglect* (physical and emotional), have found a graded relationship between the cumulative number of ACEs to which a child was exposed and a wide range of negative mental and physical health outcomes (Petruccelli et al., 2019).

Many studies have also documented such a graded relationship between ACEs and antisocial behaviour, which is characterised by violations of societal norms or laws, such as aggression, violence, and crime (Rutter et al., 1998), and a major cause of economic loss, health problems, injury, and death, particularly in low- and middle-income countries (LMICs) (Bowman et al., 2008; Matzopoulos et al., 2008). For example, using data from almost 140,000 students in the US, Duke et al. (2010) found that for every additional ACE on a cumulative score, adolescent interpersonal violence, such as fighting and weapon-carrying, increased by 35 %−144 %. Two meta-analyses reported a cumulative effect of ACEs on juvenile justice system involvement and recidivism (Graf et al., 2021; Yohros, 2022). In another meta-analysis of eight studies, Hughes et al. (2017) found that exposure to 4+ ACEs was associated with 8.1-fold increased odds of interpersonal violence, such as intimate partner violence, dating violence, and hitting someone.

A potential problem of analysing health or behavioural consequences of ACEs captured as cumulative count scores is that they assume equal weighting of each ACE in contributing to risk for the outcome. Some researchers have questioned this assumption, referring to potentially stronger effects of particularly severe types of ACEs, such as sexual abuse (Gebauer et al., 2019). Another potential limitation of focusing on cumulative ACE scores is that they may hide important interactive effects between ACEs and patterns of ACE clustering, as they are simple additive models. For example, Briggs et al. (2021) estimated that up to 40 % of the variance in ACE outcomes can be attributed to additive synergistic interactions between individual ACEs. In the National Comorbidity Survey – Replication Sample, while poverty and sexual abuse were associated with 1.1-fold and 2.9-fold increased odds of adult psychopathology, respectively, when present together, there was an additional 2.5-fold increase in the odds, on top of their individual effects. Put simply, the effects of a combination of specific ACEs may be greater than the sum of their individual contributions. It may be that for these reasons that there is often substantial heterogeneity observed in associations between multiple ACEs and health and behaviour outcomes (Hughes et al., 2017) – because of particular ACE scores reflect different ACE combinations. Overall, the ACE score, while appealing in its simplicity, may hide important differences in effects of individual ACEs as well as clusters of ACEs. Considering these inherent limitations, researchers have cautioned against using the ACE score as a screening tool and in the context of clinical decision-making (Anda et al., 2020).

A number of alternative analytical approaches have been proposed to address these limitations (Lacey and Minnis, 2020). Most prior studies considering possible differential effects of ACEs have examined each adversity independently, and most of this research was conducted outside the ACE framework; for example, child maltreatment has been consistently associated with antisocial behaviour in longitudinal studies (Braga et al., 2018, 2017). However, the *single adversity approach* does not account for potential confounding by the presence of other co-occurring adversities. To overcome this limitation, the most common method used to identify ACE clusters is *latent class analysis* (LCA). For example, in a nationally representative sample of almost 30,000 US adults, Burke et al. (2022) identified four classes of ACE exposure. The three classes with elevated levels of ACEs were more likely to engage in adult violent behaviour, compared to the low ACE reference class, indicating that even moderate ACE exposure may be harmful. Furthermore, the class characterised by high levels of both child maltreatment and household dysfunction had the highest odds of violent crime, revealing particularly harmful exposure patterns.

Another emerging method to consider how patterns of ACEs influence later outcomes is *network analysis* (Lacey, 2023), where each adversity is considered as a part of a larger system, revealing multivariate patterns of dependency between ACEs and the role of each individual ACE in the network (Borsboom et al., 2021). For example, using data from a UK birth cohort, Pollman et al. identified emotional abuse as the most central ACE, being closely linked to other adversities as well as mental health problems in early adulthood (Pollmann et al., 2022). Although such networks have been used to examine a variety of physical and mental health outcomes, there are no studies to date focusing on crime.

A limitation of current knowledge refers to the original ACE questionnaire. Although the classic ACE study used a composite measure of items from validated instruments, there was no rationale for including those specific adversities, and not others (Felitti et al., 1998). A recently published systematic review on measuring ACEs argues for the addition of new items, including community and systemic factors – dimensions which have not been previously considered as being part of the family-focused ACE framework (SmithBattle et al., 2022). Furthermore, the overwhelming majority of studies have been conducted in high-income countries (Sahle et al., 2021). This lack of evidence is particularly problematic regarding the potential effects of ACEs on crime, which may importantly depend on the cultural and social context. Almost 90 % of the world's children live in LMICs (UNICEF, 2005), often with higher rates of exposure to adversity, including interpersonal violence (WHO, 2002). Brazil, where the current study was conducted, is a middle-income country, with high inequality and a particularly high homicide rate (Igarapé Institute, 2023), with interpersonal violence being the leading cause of death in young people (Degli Esposti et al., 2023; Malta et al., 2021). Thus, children in Brazil may be exposed to a broader range and higher levels of adversity, which, in turn, may represent important vulnerabilities for later engagement in criminal or violent behaviour. Unravelling the link between ACEs and crime outcomes is therefore of utmost importance, globally and in LMICs such as Brazil specifically.

The current study aimed to examine the influence of ACEs on young adult violent and non-violent crime in a large Brazilian birth cohort. To overcome some of the limitations of current literature, we complemented the ACE scale with additional items on community- and social-level factors, and examined the relationship between ACEs and crime, using four complementary approaches: i) single adversity approach; ii) cumulative ACE risk score; iii) latent classes of ACE exposure; and iv) network analysis.

## 2 Methods

### 2.1 Participants

The 1993 Pelotas Birth Cohort is an ongoing population-based, prospective longitudinal study, investigating time trends in maternal and child health indicators and associations between early-life exposures and later life outcomes. Pelotas, Rio Grande do Sul, is located in Southern Brazil, with a population of about 340,000 people. Out of 5265 live births identified through daily hospital visits between January and December 1993, 5249 mothers (99.7 %; 50.3 % girls) agreed to participate and their children were included in the cohort. The whole cohort was assessed at birth, and when the child was 11 (87.5 %), 15 (85.7 %), 18 (81.4 %), and 22 (76.3 %) years old.^1^ Further details about the cohort can be found elsewhere (Goncalves et al., 2018; Victora et al., 2008).

### 2.2 Measures

#### 2.2.1 Adverse childhood experiences

We measured 12 ACEs at child age 11 and/or 15 years, using child self-report and/or maternal report. The items included: physical neglect; physical, emotional, and sexual abuse; domestic violence; maternal mental illness; parental divorce; ever being separated from parents; parental death; poverty; discrimination; and neighbourhood fear. Each item was dichotomised and coded as ‘yes’ if answered affirmatively at either time point and/or informant; otherwise, it was coded as ‘no’. More details on the items, their time points, and the informant used are presented in Appendix 1.

#### 2.2.2 Crime

Past-year crime was assessed in a confidential self-report questionnaire at age 22 years, using items originally developed for the Edinburgh Study of Youth Transitions and Crime, referring to behaviours committed in the 12 months preceding interview (McAra and McVie, 2010). The 14 items were previously piloted, adapted, and used in the current cohort in several prior studies (Martins et al., 2022; Murray et al., 2015, 2015). Two dichotomous outcomes were analysed: *violent* and *non-violent* crime (coded as either ‘yes’ or ‘no’). Participants were considered to have engaged in violent crime if they responded affirmatively to any of the following five criminal behaviours: stole from person with threat/force; assault; carried a weapon for fights or self-defence; used weapon; and rape. Similarly, participants were considered to have engaged in non-violent crime if they endorsed any of the following nine criminal behaviours: stole from shops/stores; damaged property; stole from vehicle; stole vehicle; sold drugs; burgled; sold stolen goods; arson; and stole from person without threat/force.

#### 2.2.3 Confounders

All confounders were measured during the perinatal assessment. Child sex (‘female’ or ‘male’), and maternal and paternal education (both used as continuous/count variables) were used as potential confounders. Furthermore, we used a health risk score at birth, which has been previously found to associate with crime in the current sample (Murray et al., 2015), including items on unplanned pregnancy (yes/no), maternal smoking during pregnancy (yes/no), maternal alcohol consumption during pregnancy (yes/no), maternal urinary tract infection during pregnancy (yes/no), intrauterine growth restriction (referring to < 10th percentile or ≥ 10th percentile for gestational age and gender, according to the reference curve developed by Kramer et al.), and premature birth (yes/no; < 37 weeks) (Kramer et al., 2001). The six items were summed, resulting in a total score ranging from 0 to 6, with higher scores indicating greater risk.

### 2.3 Statistical analysis

First, we present descriptive statistics for the total sample, and the proportions of crime outcomes for participants exposed versus those unexposed to individual ACEs.

Next, we examine univariable and multivariable associations of individual ACEs and the cumulative risk score with crime outcomes, using binary logistic regression. The ACE score was derived by summing across all items, and categorised as 0, 1, 2, 3 and 4+ ACEs, as in the original ACE Study (Felitti et al., 1998). These results are presented as odds ratios (ORs) with 95 % confidence intervals (95 % CIs). Adjusted models included child sex, maternal and paternal education, and the health risk score as confounding variables.

Third, we apply LCA to identify subgroups of participants with distinct patterns of ACE exposure. First, we estimate an *unconditional* latent class model. We compare 1- to 6-class solutions, using the following model fit indices to select the optimal class model: sample-size adjusted Bayesian Information Criterion (aBIC), which is used to reduce the risk of overfitting the model to a single sample, with lower values indicating a better model fit; and Lo-Mendell-Rubin Likelihood Ratio Test (LMR-LRT) and Bootstrapped Likelihood Ratio Test (BLRT), which are used to compare two adjacent class models, with *p*-values of < 0.05 indicating a better fit of the *k* class model compared to the *k*-1 class model. Furthermore, we consider entropy (0.40, 0.60, and 0.80 represent low, medium, and high class separation, respectively), sample size of the smallest class, and interpretability of each class (Wickrama et al., 2016). As a result of relatively poor entropy across all class solutions, we use a 1-step approach when estimating the *conditional* latent class model, which has been recommended in such cases (Bakk et al., 2013). To ensure consistency between the unconditional and conditional latent class models, we compare their item and class proportions. Again, we use binary logistic regression to examine associations between different ACE clusters and crime outcomes.

Finally, we perform network analysis using the R-package *mgm* (version 1.2–13) to estimate two undirected mixed graphical models, one for each crime outcome (Haslbeck and Waldorp, 2020). Mixed graphical models are comprised of *nodes* (i.e., observed variables) and *edges* (i.e., regression coefficients from generalised linear models, which are also called edge weights), which represent the strength of the association between two nodes after conditioning on (i.e., adjusting for) the remaining variables in the model (Borsboom et al., 2021). We estimate the networks using the least absolute shrinkage and selection operator (LASSO), which avoids overfitting by setting small edge weights to zero. The regularization parameter lambda (γ = 0.25), which determines inclusion or exclusion of edges, is selected through the extended Bayesian information criterion (EBIC). Furthermore, we include edges using the “OR” rule, which requires at least one estimate to be non-zero (i.e., node A predicts node B or node B predicts node A). Next, we visualise the networks with *qgraph* (version 1.9.2) (Epskamp et al., 2012), using multi-dimensional scaling, which allows to visually compare networks via the Procrustes algorithm (Jones et al., 2018). Using the same R-package, we assess node centrality, including strength (i.e., direct connections: the sum of edge weights of all edges connected to a node), closeness (i.e., indirect connections: the inverse of the sum of the shortest paths between one node and all other nodes), and betweenness (i.e., intermediate connections: how often is a node on the shortest path between two other nodes) (Epskamp et al., 2018). Finally, we use *bootnet* (version 1.5.3) to examine network accuracy and stability (see Appendix 2 for more details) (Epskamp et al., 2018). We compute the odds of crime as a function of ACEs, as edge parameters in mixed graphical models are more difficult to interpret. However, 95 % CIs for the ORs could not be calculated, as the conventional definition of those cannot be applied in network analysis.

To ensure a consistent sample size across approaches, we limit our sample to those with complete data on ACEs and confounders, and used a full information maximum likelihood (FIML) estimator with robust standard errors (MLR) to account for those with missing crime outcomes (Enders and Bandalos, 2001), resulting in a sample size of 3236 participants (61.6 % of the original cohort). In the main analysis, we further addressed missing data using inverse probability weighting (IPW) to minimise non-participation bias (see Appendix 3 on how weights were derived) (Seaman and White, 2013). In sensitivity analyses, we further present results without IPW, based on complete data with a sample size of 2608 (49.7 % of the original cohort). Those with complete data were less likely to be male and to have mothers who lived without a partner at baseline, when compared to the remaining sample; they also had parents with higher levels of education, older mothers at birth, and a lower health risk score. However, these differences were small to moderate in size, with Cohen's *d*s ranging between 0.08–0.21 and ORs ranging between 1.37–1.95. There were no differences for maternal alcohol consumption and maternal skin colour (see Table S3 for full details). Since missing data approaches for network analysis are not yet widely available (Borsboom et al., 2021), we limit the sample to complete cases when estimating the network models. A flow chart with details on missing data is provided in Figure S6.

Analyses related to the single adversity and cumulative ACE risk score approach were conducted in Stata, version 15.0. LCA was performed using Mplus, version 8.1. Network analysis was performed in RStudio, version 1.1.447.

## 3 Results

### 3.1 Descriptive statistics

**Fig. 1:**
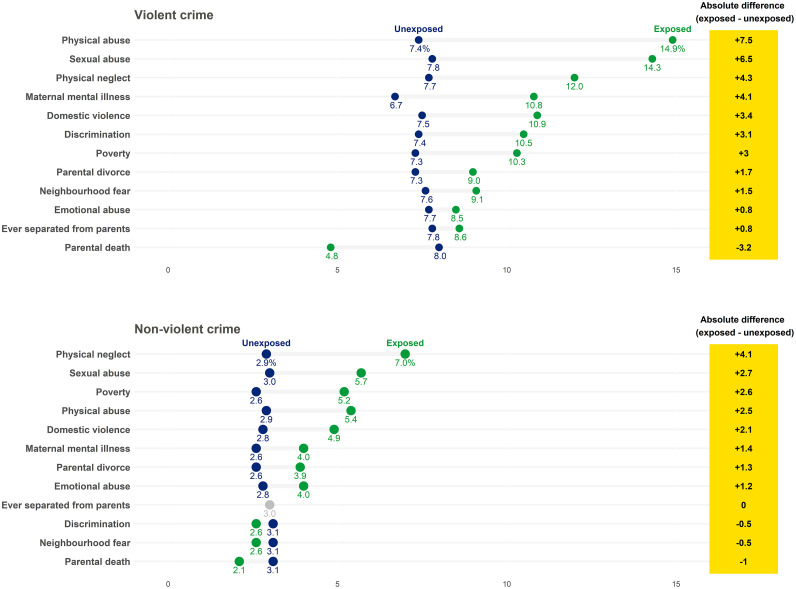
Proportions of crime outcomes for those exposed versus unexposed to specific ACEs.

***Note***. Based on available data for adverse childhood experiences and confounders (*N* = 3236). Presented in descending order from largest to smallest difference in proportions between those exposed versus those unexposed to adverse childhood experiences.

In the total sample, three-fourths (74.8 %) of participants were exposed to at least one ACE, and more than one in ten (11.0 %) reported exposure to 4+ ACEs. The most common ACE reported was parental divorce (33.9 %), and the least common ACE reported was sexual abuse (1.4 %) (see Table 1 for full details). Regarding past-year crime outcomes, 7.9 % reported having engaged in violent crime, and 3.0 % reported having committed non-violent crime. Fig. 1 presents the proportions of crime for participants exposed to individual ACEs versus those unexposed to each adversity. Participants exposed to ACEs had higher proportions of violent crime, except for parental death, which showed the opposite pattern. The largest difference was observed for physical abuse, with 14.9 % reporting violent crime among those exposed and 7.4 % among those unexposed. A similar trend was observed for non-violent crime, albeit less pronounced, with participants exposed to ACEs having higher proportions of non-violent crime. The largest difference was observed for physical neglect, with 7.0 % among those exposed and 2.9 % among those unexposed reporting non-violent crime.

**Table 1:** Adjusted models across approaches to measuring associations between adverse childhood experiences and crime outcomes.

Analytical approach n (%)	Violent crime OR (95 % CI)	Non-violent crime OR (95 % CI)
**Single adversities**^a^		
Physical neglect, *n* = 133 (4.1)	1.66 (0.86–3.19)	2.25 (0.98–5.19)
Physical abuse, *n* = 217 (6.7)	2.53 (1.56–4.09)	2.25 (1.08–4.69)
Emotional abuse, *n* = 628 (19.4)	1.52 (1.05–2.21)	1.84 (1.07–3.17)
Sexual abuse, *n* = 44 (1.4)	2.47 (0.86–7.08)	3.15 (0.70–14.12)
Domestic violence, *n* = 329 (10.2)	1.81 (1.18–2.80)	2.01 (1.08–3.76)
Maternal mental illness, *n* = 950 (29.4)	1.72 (1.26–2.34)	1.53 (0.94–2.49)
Parental divorce, *n* = 1098 (33.9)	1.20 (0.89–1.64)	1.76 (1.11–2.79)
Ever separated from parents, *n* = 252 (7.8)	1.19 (0.69–2.04)	1.29 (0.53–3.09)
Parental death, *n* = 192 (5.9)	0.62 (0.28–1.36)	0.60 (0.18–1.95)
Poverty, *n* = 594 (18.4)	1.37 (0.94–2.00)	1.96 (1.16–3.34)
Discrimination, *n* = 489 (15.1)	1.60 (1.10–2.33)	0.87 (0.43–1.75)
Neighbourhood fear, *n* = 524 (16.2)	1.38 (0.95–2.01)	0.90 (0.46–1.76)
**Cumulative ACE risk score**^a^		
0 ACE, *n* = 816 (25.2)	Ref	Ref
1 ACE, *n* = 985 (30.4)	1.19 (0.76–1.85)	1.42 (0.70–2.89)
2 ACEs, *n* = 671 (20.7)	1.71 (1.08–2.70)	1.84 (0.88–3.86)
3 ACEs, *n* = 409 (12.6)	2.21 (1.33–3.67)	2.70 (1.25–5.85)
4+ ACEs, *n* = 355 (11.0)	2.75 (1.63–4.65)	3.19 (1.43–7.13)
**Latent class analysis**^a^		
Low adversities	Ref	Ref
Child maltreatment / household challenges	3.61 (1.89–6.89)	4.37 (1.87–10.22)
Household challenges / social risks	2.39 (1.13–5.06)	1.99 (0.58–6.80)
**Network analysis**^b^		
Physical neglect		
Physical abuse	1.45	
Emotional abuse		
Sexual abuse		
Domestic violence		
Maternal mental illness	1.20	
Parental divorce		
Ever separated from parents		
Parental death		
Poverty		
Discrimination		
Neighbourhood fear		

***Note***. Associations for single adversities, the cumulative ACE risk score, and latent class analysis are based on available data for adverse childhood experiences and confounders (N = 3236) and using inverse probability weighting. Associations for network analysis are based on complete data (N = 2608) without using inverse probability weighting. For the network analysis, empty cell indicate the absence of edges; 95 % CIs for ORs in the network models were not calculated, because the sample distributions of parameters are biased and the conventional definition of 95 % CIs cannot be applied. ACE = Adverse childhood experience.

a = Adjusted for child sex, maternal education, paternal education, and a cumulative score of biological risk factors.

b = Adjusted for child sex, maternal education, paternal education, a cumulative score of biological risk factors, and all remaining childhood adverse experiences. Bold values indicate statistically significant results at *p* < .05.

### 3.2 Associations between single adversities and crime

Physical abuse, emotional abuse, and domestic violence were each associated with increased odds of both violent crime (ORs ranging between 1.52–2.53) and non-violent crime (ORs ranging between 1.84–2.25), after adjusting for confounders. For some ACEs, there was stronger evidence of associations with either violent or non-violent crime. More specifically, maternal mental illness (OR 1.72, 95 % CI 1.26–2.34) and discrimination (OR 1.60, 95 % CI 1.10–2.33) were associated with violent crime, while parental divorce (OR 1.76, 95 % CI 1.11–2.79) and poverty (OR 1.96, 95 % CI 1.16–3.34) were associated with non-violent crime. There was no or only weak evidence that the remaining ACEs were associated with either crime outcome (see Table 1 for full details). Results based on unweighted analyses showed a similar pattern of effects (see Table S4). Unadjusted models are presented in Table S5.

### 3.3 Associations between cumulative ACEs and crime

The proportions of individual ACEs for each score (i.e., exposure to 1, 2, 3, and 4+ ACEs) are presented in Figure S7. There was a dose-response relationship between the numbers of ACEs and both crime outcomes. Those exposed to 2 (OR 1.71, 95 % CI 1.08–2.70), 3 (OR 2.21, 95 % CI 1.33–3.67), and 4+ ACEs (OR 2.75, 95 % CI 1.63–4.65) were increasingly more likely to report violent crime compared to those unexposed to ACEs. Also, those exposed to 3 (OR 2.70, 95 % CI 1.25–5.85) and 4+ ACEs (OR 3.19, 95 % CI 1.43–7.13) had increasingly higher odds of engaging in non-violent crime compared to those unexposed to ACEs (see Table 1). The results were almost identical when based on unweighted analyses (see Table S4). Unadjusted models are presented in Table S5.

### 3.4 Associations between latent classes of ACEs and crime

**Fig. 2:**
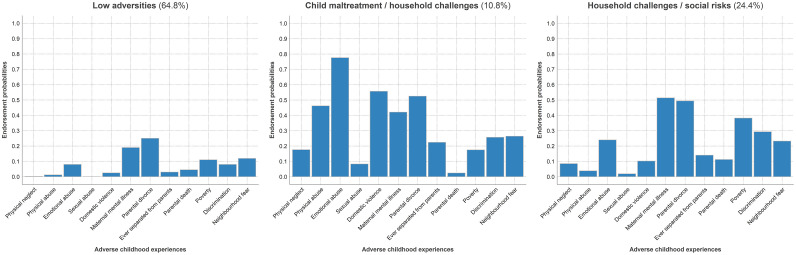
Three latent classes of adverse childhood experiences identified in the 1993 Pelotas Birth Cohort.

***Note***. Based on available data for adverse childhood experiences and confounders (*N* = 3236). Dark blue segments represent positive probabilities (i.e., being exposed to adverse childhood experiences)

Fig. 2 shows the three ACE classes identified in the LCA (for details on how the optimal class model was selected see Appendix 4 and Table S6). Class 1 (64.8 %) was characterised by generally *low adversities*. In this class, the probabilities of ACEs ranged between 0 % and 11.9 %, except for parental divorce (25.0 %) and maternal mental illness (19.0 %). By contrast, participants in class 2 (10.8 %) were exposed to a broader range of ACEs, including particularly high rates of exposure to *child maltreatment* (up to almost 80 %) and *household challenges* (up to almost 60 %). Finally, class 3 (24.4 %) was characterised by exposure to *household challenges and social risks*, with higher proportions of maternal mental illness (51.4 %) and parental divorce (49.4 %), in addition to raised levels of poverty, discrimination, and neighbourhood fear, with proportions ranging between 23.2 % and 38.2 %.

We examined associations between the latent classes of ACEs and crime outcomes, using the low adversities class as the reference group. After adjusting for confounders, the child maltreatment and household challenges class (OR 3.61, 95 % CI 1.89–6.89) and the household challenges and social risks class (OR 2.39, 95 % CI 1.13–5.06) had higher odds for violent crime compared to the low adversities class. For non-violent crime, the child maltreatment and household challenges class had particularly high odds compared to the low adversities class (OR 4.37, 95 % CI 1.87–10.22). The full results are presented in Table 1. Results were similar without IPW and when based on complete case analysis (see Tables S8 and S9). Unadjusted models are presented in Table S5.

### 3.5 Network models of ACEs and crime

**Fig. 3:**
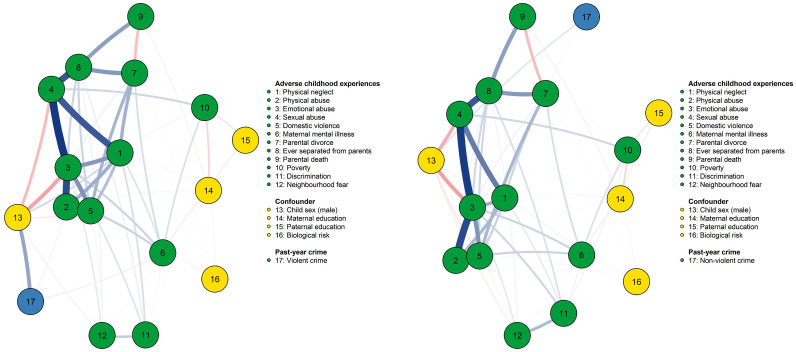
Network models including adverse childhood experiences, confounders, and violent (left panel) and non-violent crime (right panel).

***Note.*** Based on the Procrustes algorithm, where node placement directly corresponds to similarities and differences between two networks. Blue edges indicate positive associations. Red edges indicate negative associations. Violent crime shows edges with physical abuse, maternal mental illness, and child sex (male). Non-violent crime shows edges with parental divorce and child sex (male).

Fig. 3 shows the results of the network models of ACEs and crime outcomes. Violent crime (left panel) and non-violent crime (right panel) are marked by the blue circle (number 17). In the network for violent crime, physical abuse (OR 1.45) and maternal mental illness (OR 1.20) were associated with increased odds of violence, conditioning on all other variables in the model (see Table 1). Non-violent crime was associated with parental divorce, after conditioning on the remaining variables. Again, the network models were estimated using the “OR” rule, which required either node A to predict node B or node B to predict node A to be included in the model. Upon further examination, only non-violent crime predicted parental divorce (OR 1.15) but not vice versa. Thus, the odds of non-violent crime as a function of parental divorce could not be calculated. Notably, some edges were absent across models, for example, between domestic violence and crime outcomes, suggesting conditional independence between these two variables given all remaining variables in the networks.

Considering the interrelationships between ACEs, there were particularly strong connections (here defined as edge weights ≥ 0.60) between different types of child maltreatment, including physical and emotional abuse, physical abuse and domestic violence, emotional and sexual abuse, as well as and sexual abuse and ever being separated from parents; this was true across both networks — for violent and non-violent crime. The absence of some edges, for example, between physical abuse and maternal mental illness again indicates that these ACEs are conditionally independent.

**Fig. 4:**
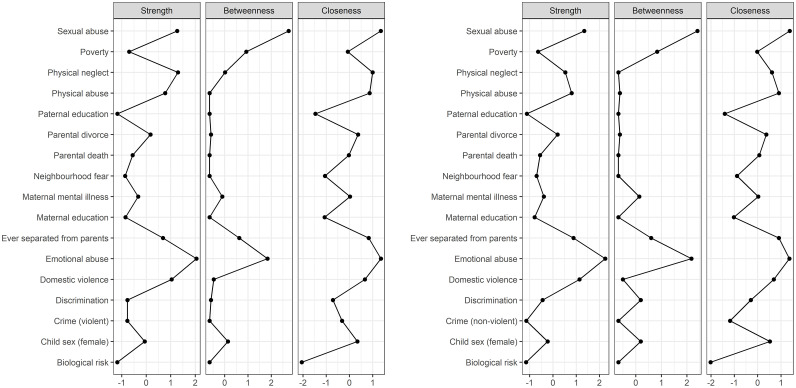
Centrality measures for violent (left panel) and non-violent crime (right panel).

***Note.*** Ordered alphabetically in reverse order from top to bottom. Strength = direct connections (i.e., the sum of edge weights of all edges connected to a node). Closeness = indirect connections (i.e., the inverse of the sum of the shortest paths between one node and all other nodes). Betweenness = intermediate connections (i.e., often is a node on the shortest path between two other nodes)

Emotional abuse and sexual abuse were the most central nodes in both networks. While emotional abuse was most strongly associated with other nodes in the network (strength), sexual abuse was most often on the shortest path between *two* other nodes (betweenness). Both types of abuse showed the shortest paths to *all* other nodes (closeness). Fig. 4 presents the centrality measures for all variables in the models. See Appendix 2 for details on network accuracy and stability.

## 4 Discussion

The current study examined longitudinal associations between ACEs and violent and non-violent crime in young adulthood, using data from a large Brazilian birth cohort using four analytical approaches: a single adversity approach, cumulative risk, latent classes of ACE exposure, and network analysis. We found that ACEs – considered individually, cumulatively, and as clusters – were associated with increased odds of violent and non-violent crime. Although there was a cumulative increase in the odds for crime according to the number of ACEs to which participants were exposed, some individual and combinations of ACEs showed particularly strong and robust effects, which were not captured by the simple ACE score. Furthermore, some ACEs were more strongly linked to each other than others, and may influence crime through different pathways as part of a larger system of co-occurring adversities.

In line with previous research (Hughes et al., 2017), our findings provide further support for an important cumulative effect of ACEs on crime — there was a roughly stepwise progression in risk, with exposure to 2+, 3+, and 4+ ACEs being associated with up to 3-fold increased odds for violent and non-violent crime, compared to no ACE. Exposure to only 1 ACE, on average, was not associated with crime, but it is critical to remember that the ACE score does not differentiate between types of adversity, and effects of a single ACE with particularly strong effects may be masked in this ACE score of “*any* 1 ACE”. Importantly, when examining each ACE individually, and as part of the network models, several specific ACEs were strongly associated with crime. Across the two approaches, there was consistent evidence that physical abuse and maternal mental illness had particularly robust associations with violent crime, and parental divorce was strongly associated with non-violent crime.

The way multiple ACEs cluster and then impact on crime is probably best represented by the results from the LCA. These showed that experiencing multiple household challenges (such as maternal mental illness and parental divorce) and social risks (such as poverty and discrimination) associate with mainly violent, but not non-violent, crime. These findings are in line with Burke et al. (2022), who showed that all elevated ACE classes, irrespective of severity and type of adversity, were associated with increased odds of adult violent behaviour. However, participants with multiple ACEs involving either child maltreatment or domestic violence had the highest odds for these crime outcomes. This is supported by a long-standing child maltreatment literature, which shows that those exposed to multiple types of child abuse and neglect are at highest risk of adverse outcomes (Debowska et al., 2017; Rivera et al., 2018). A recent scoping review on latent classes of ACE exposure including 58 studies provides further evidence that the high/multiple ACEs class is associated with the worst outcomes, with child maltreatment exerting particularly strong effects (Wang et al., 2023). When further assessing how and which ACEs are linked to each other, there were particularly strong associations between different types of child maltreatment (physical, emotional, and sexual abuse, and domestic violence), with emotional and sexual abuse being the most central nodes in both networks. These results support previous research on the interrelatedness of ACEs (Dong et al., 2004), and further illustrates the limitations of the single adversity approach, particularly for types of child abuse and neglect, which may not occur in isolation. Furthermore, these findings are partially supported by another study using data from a UK birth cohort, which found emotional abuse most strongly connected to other ACEs and acting as a bridge between clusters of ACEs and adult mental health problems (Pollmann et al., 2022).

Childhood adversity and trauma have been proposed as key *transdiagnostic* risk factors (McLaughlin, 2016; McLaughlin et al., 2020), with evidence from a large number of systematic reviews and meta-analyses, showing elevated risk for a wide range of health outcomes (Sahle et al., 2021). However, less is known about the issue of specificity versus generality regarding the effects of ACEs on different types of offending (i.e., whether ACE exposure might be associated with increased risk of specific crime types or patterns through time, or a more general criminal propensity). Using data from justice-involved youth in the US, cumulative ACE exposure was particularly associated with early-onset, chronic, and violent offending (Baglivio et al., 2015; Fox et al., 2015). The current study found evidence for a dose-response relationship between ACEs and both violent and non-violent crime, albeit the association was slightly more pronounced for violent crime. Some individual ACEs were associated with both crime outcomes, whereas others were associated with either violent or non-violent crime, again, providing evidence for both generality and specificity. Furthermore, while the class with child maltreatment and household challenges was associated with both crime outcomes, indicating non-specific associations, the class with household challenges and social risks was mainly associated with violent, but not non-violent, crime, suggesting some specificity. However, comparisons between effect sizes should be made cautiously, as no post-hoc contrasts were conducted. The issue of potential specificity of effects of ACEs on certain types or patterns of crime warrants further research, using more varied forms of offending.

More than seven out of 10 children in this sample were exposed to at least one ACE, and almost one in four reported exposure to 3+ ACEs. The prevalence of ACEs was examined in a systematic review of studies using the ACE International Questionnaire (Pace et al., 2022). Across 63 community samples (77 % from Asia and Africa), the average prevalence of experiencing at least one ACE was 75 %. Considering the lack of a consistent definition as to what constitutes childhood adversity, wording of items to measure them, different item availability across samples, and varying age ranges across studies, researchers need to carefully balance between generalisability (i.e., using comparable measures across studies) and specificity (i.e., adapting measures to specific cultural and social contexts).

There was some evidence of negative confounding across analytical approaches, as effect sizes for some associations were slightly larger in adjusted models compared to unadjusted models. Child sex had associations in opposite directions with the exposure and outcome, which may explain this pattern of results (see Table S10).

The following limitations should be considered when interpreting the findings. First, there was some attrition over time, with some differences on sociodemographic characteristics between those who remained in the study and those who dropped out. For the regression models and LCA, we limited the sample to those with complete data on ACEs and confounders, but used FIML to incorporate those with missing data on crime (under the missing-at-random assumption). Additionally, we applied IPW to reduce the potential bias of missing data by allocating sample weights to the analysis sample. However, these approaches were not applicable to the network models, which were based on complete cases. Second, we assessed ACEs up to age 15 years, as opposed to covering up to 18 years as in most previous studies. Furthermore, some ACEs were only measured at age 11 years (e.g., maternal mental illness) or were limited to experiences in the past six months (physical abuse). Finally, the wording of questions may have influenced responses, particularly the item on discrimination, which asked about various forms of prejudice rather than specific discriminatory acts. Thus, although the study has the advantage of not being reliant on retrospective adult reports on childhood ACEs (as in many prior studies), the prevalence of ACEs may be underestimated by these measurement issues. Another limitation is that both LCA and network analysis are empirically-driven methods, which may yield different results across samples, reducing potential generalisability. Our latent class model showed relatively poor entropy, particularly between the two elevated ACE classes (see Table S7). Therefore, we decided to use a 1-step approach, which has been shown to be most appropriate for models with low entropy (Bakk et al., 2013). Nevertheless, comparisons between the elevated classes should be made with caution. Although ACE clusters have been widely studied, distinct patterns of ACE exposure are not always identified, as has been shown in similar birth cohorts such as the UK-based Millennium Cohort Study (Bevilacqua et al., 2021). Finally, the current study did not consider other factors involved in crime such as delinquent peers, school performance, employment problems, and mental health problems (Basto-Pereira and Farrington, 2022), which could be considered as potential mechanisms linking ACEs and crime in future studies (Hales et al., 2022).

Researchers, practitioners, and policymakers should be aware that ACEs are common in young adults engaging in criminal behaviour, and that they may present diverse ACE exposure profiles. Thus, there is value in screening youth in contact with the criminal justice system for ACEs; however, similar to other researchers (Anda et al., 2020), we caution against using a cumulative risk score for this purpose. Although we observed a relatively stable increase across all items when comparing exposure to 1, 2, 3, and 4+ ACEs on the cumulative score (see Figure S7), there was also strong evidence for item-specific interrelatedness and clustering. Researchers are advised to further examine the effectiveness of programmes to reduce maltreatment and other forms of violence against children, which may reduce the risk of later crime outcomes. These may focus on parenting interventions targeting child maltreatment, for example, which have shown effectiveness in reducing antisocial behaviour and delinquency (Knerr et al., 2013; Piquero et al., 2016). Alternatively, prevention programmes supported by an understanding of ACEs may target youth already involved in crime. While there is some evidence of trauma-informed care in the juvenile justice setting (Zettler, 2020), more research is needed from community-based samples and older populations.

In conclusion, some ACEs are more strongly connected with each other than others, and show different patterns of clustering. Both individually and in combination, they may increase the odds for later violent and non-violent crime in the Brazilian context. Although an overall dose-response relationship was observed between ACEs and crime, there is also evidence that some ACEs have particularly strong effects, both when considered individually, and when grouped with other ACEs. Thus, researchers, clinicians, and policymakers need to further evaluate how information on ACEs can most effectively prevent adverse consequences through the life-course, including later criminal behaviour.

## Ethical considerations

All assessments were approved by the Federal University of Pelotas Medical School Research Ethics Committee. Mothers were informed of all follow-up procedures, the study objectives, the voluntary nature of their participation, and their right not to participate, to answer specific questions, and to the confidentiality of their information.

## Data statement

Applications to use the data can be made by contacting the researchers of the 1993 cohort (see http://www.epidemio-ufpel.org.br/site/content/faculty/ for a list of key faculty members) and completing the application form (http://www.epidemio-ufpel.org.br/site/content/studies/formularios.php). A list of administered questionnaires at each timepoint can be accessed online (http://www.epidemio-ufpel.org.br/site/content/coorte_1993-en/questionnaires.php). Researchers with successful applications will receive a dataset including the requested variables and unique participant IDs.

## Financial support

This article is based on data from the study “Pelotas Birth Cohort, 1993” conducted by the Postgraduate Program in Epidemiology at *Universidade Federal de Pelotas* with the collaboration of the Brazilian Public Health Association (ABRASCO). From 2004 to 2013, the 10.13039/100010269Wellcome Trust supported the 1993 birth cohort study. The European Union, the National Support Program for Centers of Excellence (PRONEX), the Brazilian National Research Council (CNPq), and the Brazilian Ministry of Health supported previous phases of the study. The 22-year follow-up was supported by the Science and Technology Department/Brazilian Ministry of Health, with resources transferred through the Brazilian National Council for Scientific and Technological Development (CNPq), grant 400943/2013–1. Lastly, analyses were supported in part by the 10.13039/501100002322Coordenação de Aperfeiçoamento de Pessoal de Nível Superior*—Brasil* (CAPES)—Finance Code 001, and the 10.13039/100000865Bill and Melinda Gates Foundation (OPP1164115). This research was funded in whole, or in part, by the 10.13039/100010269Wellcome Trust [Grant number 210735_A_18_Z]. Gemma Hammerton was supported by a Sir Henry Wellcome Postdoctoral Fellowship (grant number 209138/Z/17/Z). For the purpose of open access, the author has applied a CC BY public copyright licence to any Author Accepted Manuscript version arising from this submission.

## CRediT authorship contribution statement

**Andreas Bauer:** Writing – original draft, Visualization, Methodology, Investigation, Formal analysis, Conceptualization. **Rafaela Costa Martins:** Writing – original draft, Methodology, Investigation, Formal analysis, Data curation, Conceptualization. **Gemma Hammerton:** Writing – review & editing, Methodology, Formal analysis, Conceptualization. **Maurício Scopel Hoffmann:** Writing – review & editing, Methodology, Formal analysis, Conceptualization. **Andressa Souza Cardoso:** Writing – review & editing, Methodology, Investigation, Data curation, Conceptualization. **Camila Colvara:** Writing – review & editing, Methodology, Investigation, Data curation, Conceptualization. **Clarissa Fialho Hartmann:** Writing – review & editing, Methodology, Investigation, Data curation, Conceptualization. **Gabriel Calegaro:** Writing – review & editing, Methodology, Investigation, Data curation, Conceptualization. **Luciana Rodrigues Perrone:** Writing – review & editing, Methodology, Investigation, Data curation, Conceptualization. **Nilvia Aurélio:** Writing – review & editing, Methodology, Investigation, Data curation, Conceptualization. **Ana M.B. Menezes:** Conceptualization, Data curation, Investigation, Methodology, Writing – review & editing. **Joseph Murray:** Writing – original draft, Supervision, Project administration, Methodology, Funding acquisition, Data curation, Conceptualization.

## Declaration of competing interest

None.

## Appendix Supplementary materials

Image, application 1

Image, application 2

Image, application 3

Image, application 4

Image, application 5

## Footnotes

1 At ages 1, 3, 6, 12 and 48 months, follow-up visits were limited to sub-samples (see cohort profile for more details).
