# Supplementary figures and images for: Adverse childhood experiences and crime outcomes in early adulthood: A multi-method approach in a Brazilian birth cohort
Source: Psychiatry Res. 2024 Apr;334:115809. doi: 10.1016/j.psychres.2024.115809 (PMC10985840; doi:10.1016/j.psychres.2024.115809)

● Bootstrap mean ● Sample

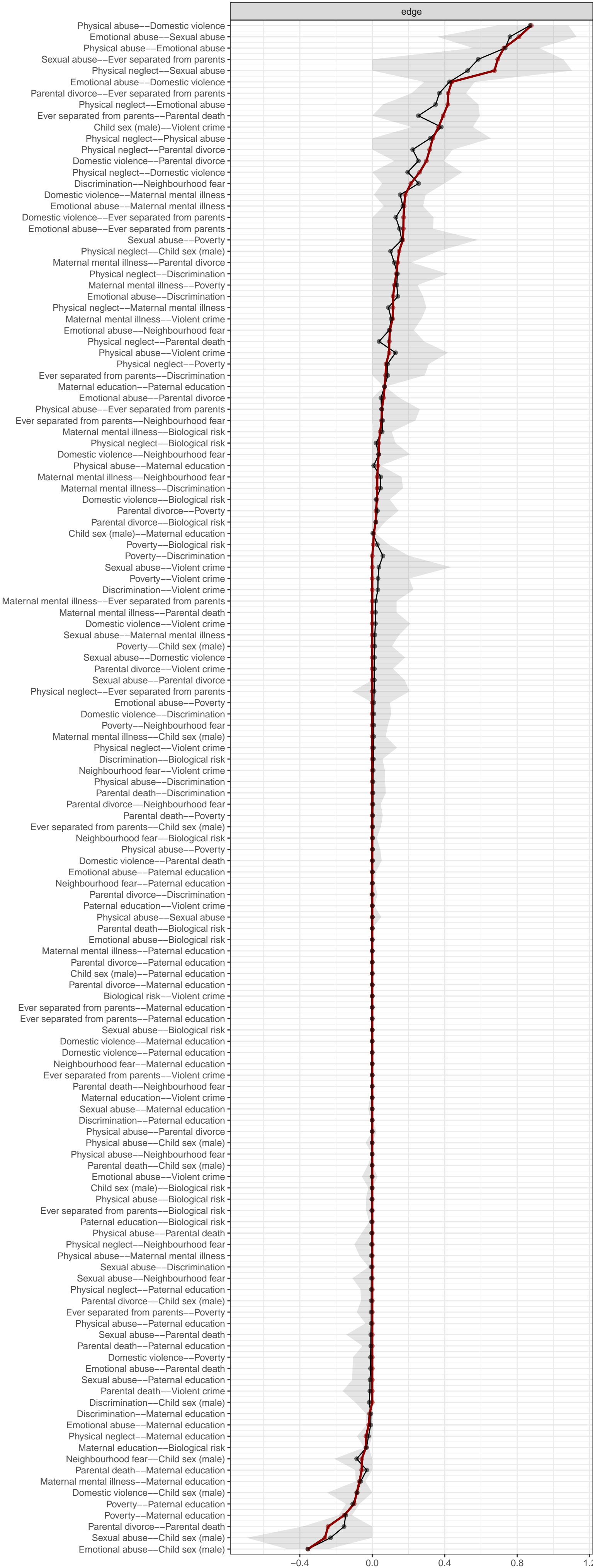

Supplement: Supplementary file 1 [file mmc1.pdf]

● Bootstrap mean    ● Sample

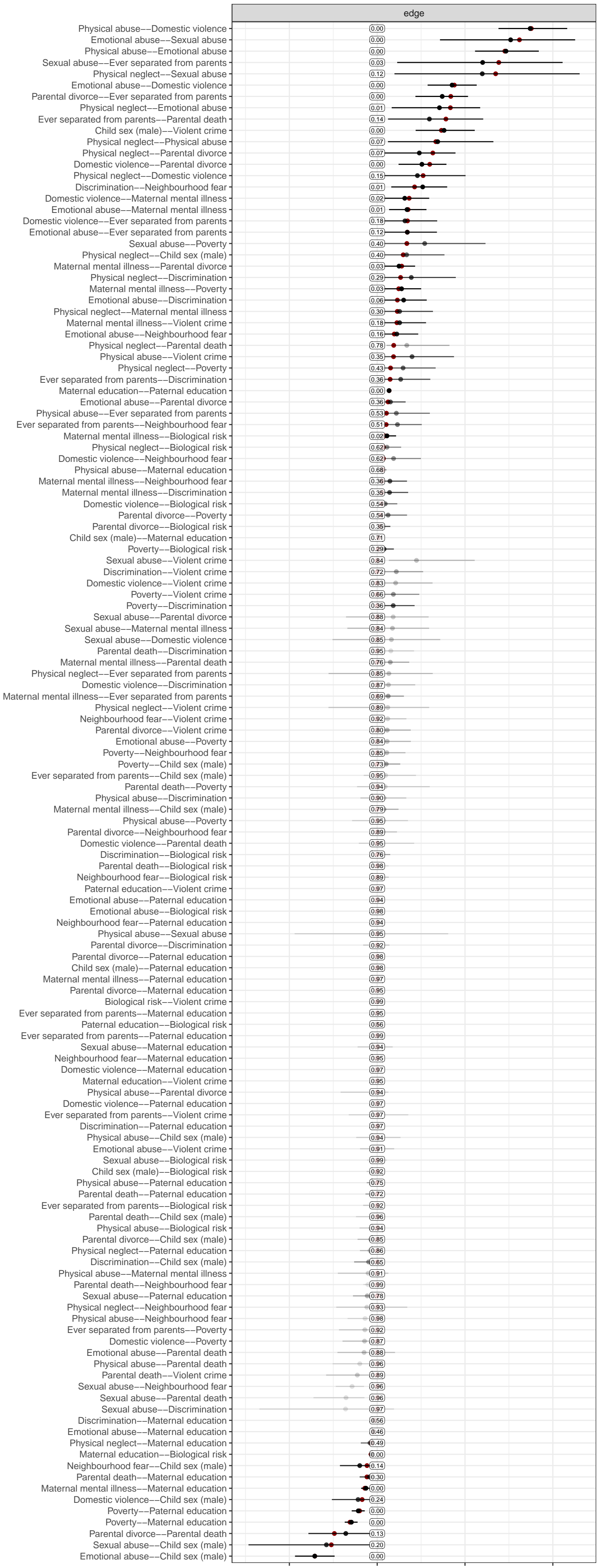

Supplement: Supplementary file 2 [file mmc2.pdf]

● Bootstrap mean ● Sample

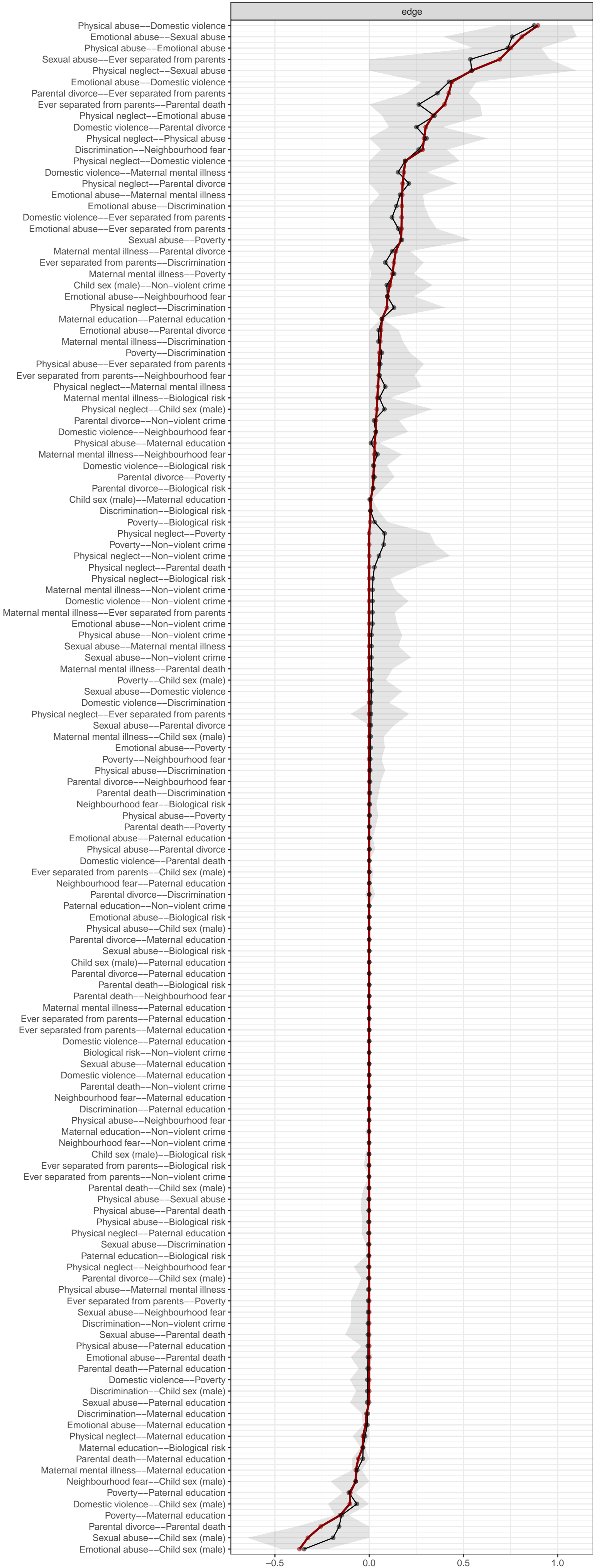

Supplement: Supplementary file 3 [file mmc3.pdf]

● Bootstrap mean    ● Sample

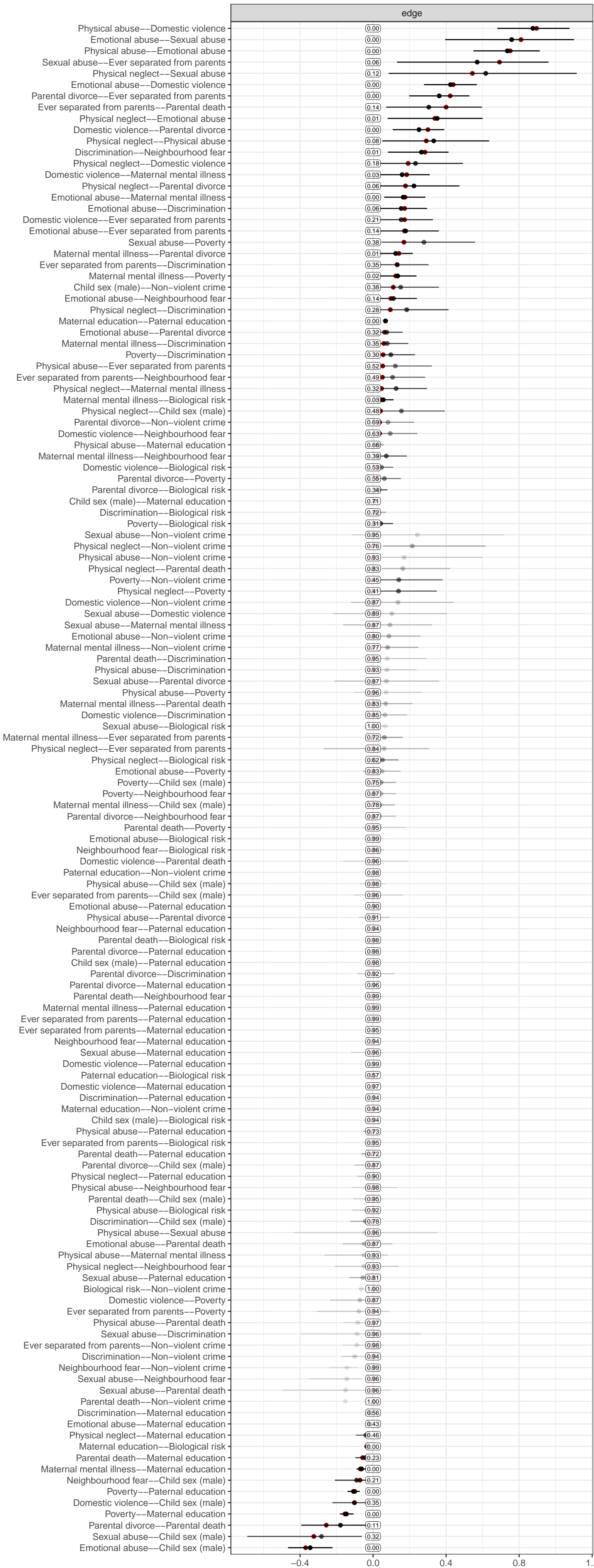

Supplement: Supplementary file 4 [file mmc4.pdf]
