# Supplementary material for: Adverse childhood experiences and crime outcomes in early adulthood: A multi-method approach in a Brazilian birth cohort
Source: Psychiatry Res. 2024 Apr;334:115809. doi: 10.1016/j.psychres.2024.115809 (PMC10985840; doi:10.1016/j.psychres.2024.115809)
Supplement: Supplementary file 5 [file mmc5.docx]

**Supporting information**

**Adverse childhood experiences and crime outcomes in early adulthood:**

**a multi-method approach in a Brazilian birth cohort**

Andreas Bauer, Rafaela Costa Martins, Gemma Hammerton, Maurício Scopel Hoffmann, Andressa Souza Cardoso, Camila Colvara, Clarissa Fialho Hartmann, Gabriel Calegaro, Luciana Rodrigues Perrone, Nilvia Aurélio, Ana M. B. Menezes, Joseph Murray

**Appendix 1** Details on how adverse childhood experiences were measured

| **Table S1** Items used to measure adverse childhood experiences, their time points, and the informant used | | |  |
| --- | --- | --- | --- |
| **Adverse childhood experiences** | **Item(s)** | **Age(s)** | **Informant** |
| Physical neglect | *Have you ever not had enough food at home or had to wear dirty/worn clothes because you had no others?* | 15 | Child |
| Physical abuse^a^ | *In the past 6 months, has an adult of your family or someone who was looking after you hit you in a way that left you hurt or bruised?* | 11, 15 | Child |
| Emotional abuse^b^ | *Have you ever thought or felt that your parents did not want you to have been born? Have you ever thought or felt that someone in your family hates you?* | 15 | Child |
| Sexual abuse | *Has anyone ever tried to do sexual things to you against your will, threatening or hurting you?* | 15 | Child |
| Domestic violence | *Have there ever been fights with physical assault in your household between adults or has an adult ever assaulted a child or adolescent?* | 15 | Child |
| Maternal mental illness | A score of ≥ 8 on the SRQ-20 (see details below) | 11 | Mother |
| Parental divorce^b^ | *Are your parents separated? Have you and the natural father of your child ever get divorced?* | 15 | Child |
|  |  |  | Mother |
| Ever separated from parents | *Have you ever been separated from your parents to be taken care of by someone else?* | 15 | Child |
| Parental death^a^ | *Is your natural mother/father alive?*  *Is the natural mother/father alive?* | 11, 15 | Child |
|  |  |  | Mother |
| Poverty | Measured as change in family income, coded as ‘always poor’ (lower tercile of family income at both time points) and ‘not always poor’ (middle/upper tercile at either time point) | birth, 11 | Mother |
| Discrimination | *Since last month, have you felt discriminated or disadvantaged because of your skin colour/race, religion, wealth/poverty, illness/physical disability?* | 11 | Child |
| Neighbourhood fear | *Have you ever been in fear of living in your neighbourhood?* | 11 | Child |
| ***Note.*** ^a^ = If answered affirmatively at either time point. ^b^ = If either item was answered affirmatively. SRQ = Self-Report Questionnaire. | | | |

We used the Self-Report Questionnaire (SRQ-20) to assess maternal mental illness. The SRQ-20 was developed by the WHO to screen for common mental disorders in the past month, including depressive, anxiety, and psychosomatic disorders (Beusenberg et al., 1994). At child age 11 years, mothers completed the SRQ-20, which consists of 20 items, coded as ‘yes’ (1) or ‘no’ (0), resulting in a total score of 0-20. Similar to previous studies using the same sample (Gomes et al., 2019), we used the recommended cut-off (i.e., a score of ≥ 8) (Mari & Williams, 1986), which has been shown to be a conservative estimate of psychiatric disorders (Barreto do Carmo et al., 2017).

**Appendix 2** Details on how network accuracy and stability were examined

We assessed edge-weight accuracy, using non-parametric bootstrapping with 500 samples. To examine the stability of centrality indices, we used case-dropping bootstrapping with 500 samples. We additionally computed the correlation stability (CS) coefficient to quantify centrality stability (Epskamp et al., 2018). More specifically, the CS-coefficient represents the maximum proportion of participants that can be removed from the sample to maintain a correlation of ≥ 0.7 between the centrality measures of the estimated total sample and a subset of the sample. According to a simulation study, the CS-coefficient should be ≥ 0.25 and ideally > 0.50 to allow interpretation of differences in node centrality (Epskamp et al., 2018).

Betweenness showed particularly poor stability, whereas strength and closeness were more stable in both models (see Figure S1). More specifically, when using case-dropping bootstrapping (i.e., dropping participants from the sample), the order of node betweenness coefficients may change. Furthermore, the *CS*-coefficients indicated that strength was the most stable centrality measure (violent crime, *CS*(cor = 0.7) = 0.44; non-violent crime, *CS*(cor = 0.7) = 0.59), whereas closeness (violent crime, *CS*(cor = 0.7) = 0.36; non-violent crime, *CS*(cor = 0.7) = 0.00) and betweenness (violent crime, *CS*(cor = 0.7) = 0.00; non-violent crime, *CS*(cor = 0.7) = 0.05), were not stable when participants are removed from the sample. Thus, particularly the order of node betweenness and closeness should be interpreted with caution. Nevertheless, both network models showed high stability, with the majority of edges in the network being also included in most bootstrapped models (see Figures S2-S5 for more details).

**Figure S1** Average correlations between centrality indices, including betweenness, closeness, and strength, between the estimated (original) network and networks with participants removed from the analysis


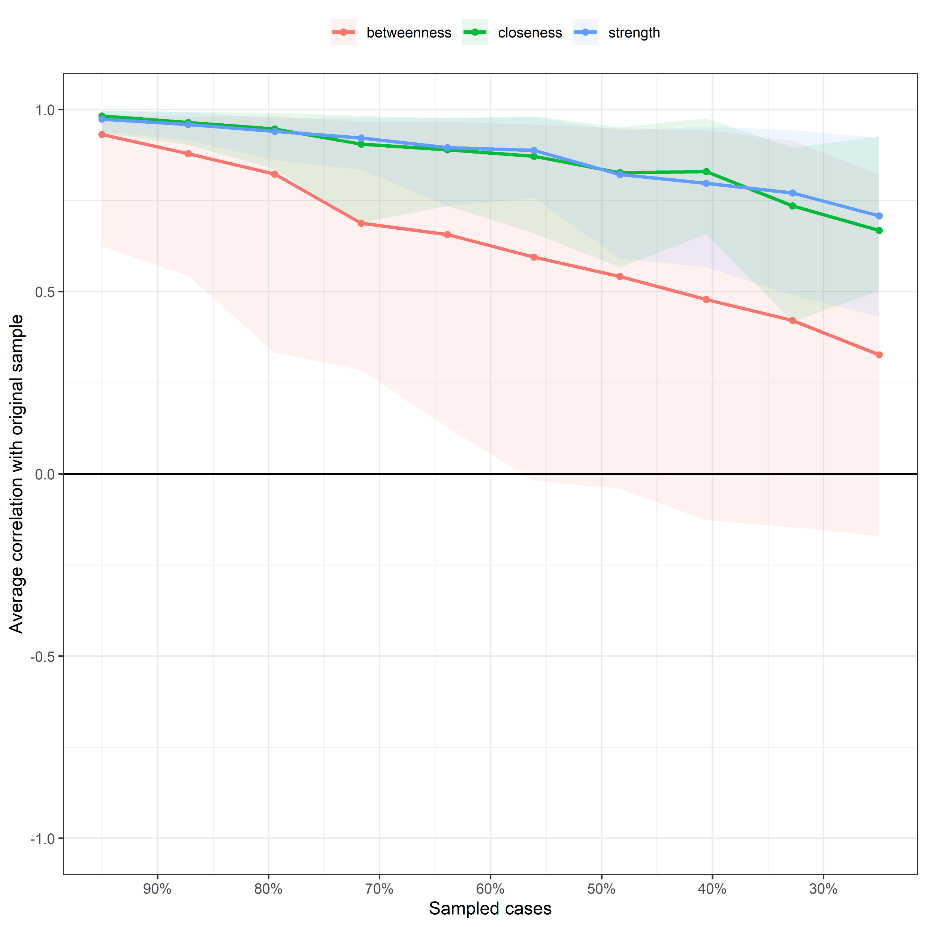

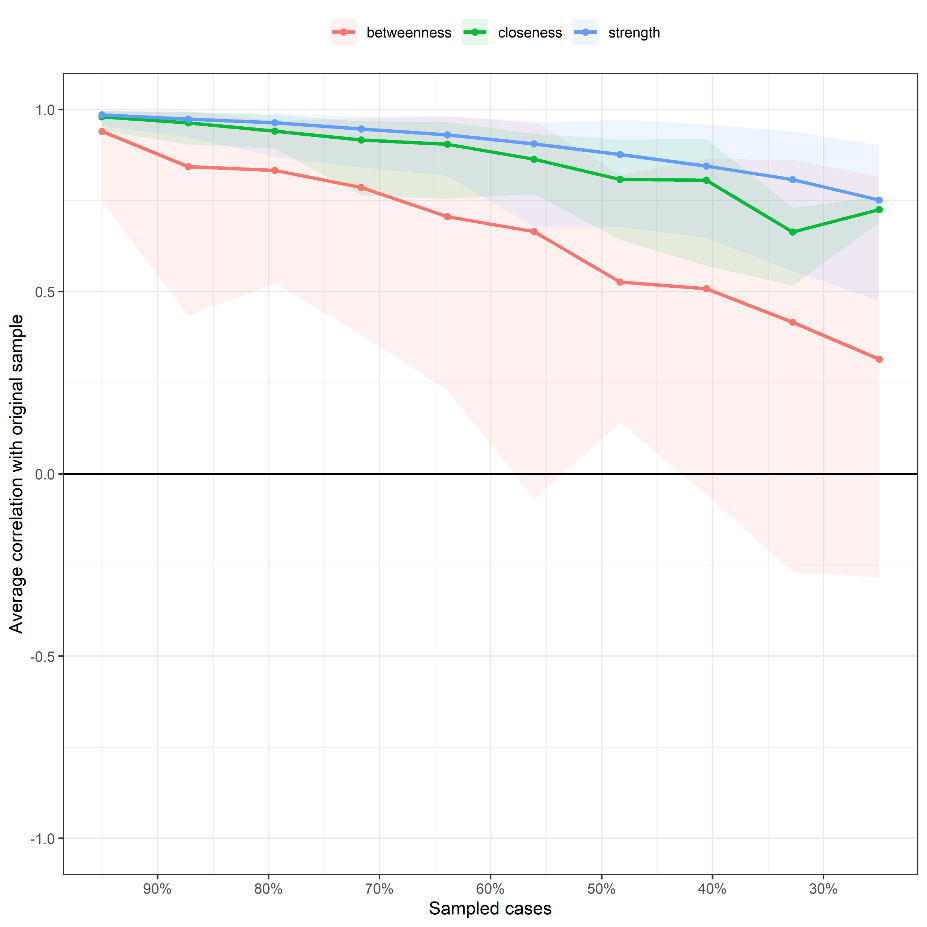


**Note.** Lines indicate mean correlation coefficients of centrality indices between the estimated sample and a subset of the sample (e.g., 90% of sampled cases). The coloured areas represent the 95% quintiles of mean correlation coefficients.

**Figure S2** Bootstrapped confidence intervals of estimated edge-weights for the network on violent crime

***Note.*** Based on non-parametric bootstrapping with 500 samples. The y-axis shows all possible 136 edges in the network; the x-axis shows the strength of the edge weights. The red line represents the point estimates of all edges in the sample, the black line and grey area represent the mean estimates of all edges across the 500 bootstrap samples and 95% bootstrapped quintiles, respectively. Edges are ordered by strength, from +1 (i.e., positive relationship) to -1 (i.e., negative relationship).

**Figure S3** Bootstrapped confidence intervals on how often an edge was included in the network on violent crime

***Note.*** Based on non-parametric bootstrapping with 500 samples. The y-axis shows all possible 136 edges in the network; the x-axis shows the strength of the edge weights. The red dots represent the point estimates of all edges in the sample, the black dot and line represent the mean estimates of all edges across the 500 bootstrap samples and 95% bootstrapped quintiles, respectively. The box indicates how often the parameter was set to zero, with 0 indicating that the edge was always included and 1 indicating that the edge was never included. Edges are ordered by strength, from +1 (i.e., positive relationship) to -1 (i.e., negative relationship).

**Figure S4** Bootstrapped confidence intervals of estimated edge-weights for the network on non-violent crime

***Note.*** Based on non-parametric bootstrapping with 500 samples. The y-axis shows all possible 136 edges in the network; the x-axis shows the strength of the edge weights. The red line represents the point estimates of all edges in the sample, the black line and grey area represent the mean estimates of all edges across the 500 bootstrap samples and 95% bootstrapped quintiles, respectively. Edges are ordered by strength, from +1 (i.e., positive relationship) to -1 (i.e., negative relationship).

**Figure S5** Bootstrapped confidence intervals on how often an edge was included in the network on non-violent crime

***Note.*** Based on non-parametric bootstrapping with 500 samples. The y-axis shows all possible 136 edges in the network; the x-axis shows the strength of the edge weights. The red dots represent the point estimates of all edges in the sample, the black dot and line represent the mean estimates of all edges across the 500 bootstrap samples and 95% bootstrapped quintiles, respectively. The box indicates how often the parameter was set to zero, with 0 indicating that the edge was always included and 1 indicating that the edge was never included. Edges are ordered by strength, from +1 (i.e., positive relationship) to -1 (i.e., negative relationship).

**Appendix 3** Information on how inverse probability weights were derived

We examined the following perinatal variables as potential inverse probability weighting (IPW) indicators: child sex (‘female’ or ‘male’), maternal smoking (‘no’ or ‘yes’), maternal alcohol consumption (‘no’ or ‘yes’), mother living with partner (‘yes’ or ‘no’), maternal skin colour (‘White’ or ‘non-White’), and maternal age, maternal education, and paternal education, which were all used as continuous variables. Missing data on IPW indicators were singly imputed as the mode (for binary variables) or the mean (for continuous variables). Missingness ranged between 0% and 0.1%, except for paternal education, which showed larger amounts of missingness (7.5%). IPW indicators were selected if they were independently associated with missingness *and* independently associated with *either* the exposure (i.e., the low adversities class vs. the two elevated classes) or any crime outcome (additionally adjusting for the exposure). In sum, child sex, mother living with partner, and maternal education met these criteria and were selected as IPW indicators. Weights ranged from 1.2 to 3.0. Descriptive statistics for the three IPW indicators for those included in the analysis sample and those missing from the analysis are presented in Table S2.

| **Table S2** Associations between indicators to derive the inverse probability weights and inclusion in the analysis sample | | | | |
| --- | --- | --- | --- | --- |
| **IPW indicators** | **Analysis sample** (*N*=3,236) | **Missing** (*N*=2,013) |  |  |
|  | % (*n*)  or Mean (SD) | % (*n*)  or Mean (SD) | OR (95% CI) or Cohen’s *d* | *p*-value |
| **Child sex** |  |  |  |  |
| *Male* | 48.2 (1561) | 51.8 (1024) | 1.15 (1.03-1.29) | = .013 |
| **Mother living with partner** |  |  |  |  |
| *No* | 8.5 (274) | 18.6 (375) | 2.47 (2.09-2.94) | < .001 |
| **Maternal education** | 7.0 (3.5) | 6.3 (3.4) | 0.20 | < .001 |
| ***Note.*** Based on singly imputed data, *N*=5,249. IPW = Inverse probability weighting. | | | | |

| **Table S3** Comparisons between complete cases and the remaining sample on confounders, IPW indicators, and other sociodemographic characteristics | | | | |
| --- | --- | --- | --- | --- |
|  | **Complete cases** (*N*=2,608) | **Remaining sample** (*N*=2,641) |  |  |
|  | % (*n*)  or Mean (SD) | % (*n*)  or Mean (SD) | OR (95% CI) or Cohen’s *d* | *p*-value |
| **Child sex** |  |  |  |  |
| *Male* | 45.7 (1,191) | 53.5 (1,412) | 1.37 (1.23-1.53) | < .001 |
| **Maternal education** | 7.1 (3.5) | 6.4 (3.7) | 0.21 | < .001 |
| **Paternal education** | 7.0 (3.5) | 6.7 (3.6) | 0.09 | = .001 |
| **Health risk score** | 1.5 (1.1) | 1.6 (1.1) | 0.12 | < .001 |
| **Mother living with partner** |  |  |  |  |
| *No* | 8.8 (230) | 15.9 (419) | 1.95 (1.64-2.32) | < .001 |
| **Maternal alcohol consumption** |  |  |  |  |
| *Yes* | 5.0 (130) | 5.2 (137) | 1.04 (0.81-1.35) | = .738 |
| **Maternal skin colour** |  |  |  |  |
| *Non-White* | 21.9 (570) | 23.5 (619) | 1.10 (0.96-1.25) | = .166 |
| **Maternal age** | 26.3 (6.3) | 25.8 (6.5) | 0.08 | = .004 |

**Figure S6** Flow chart


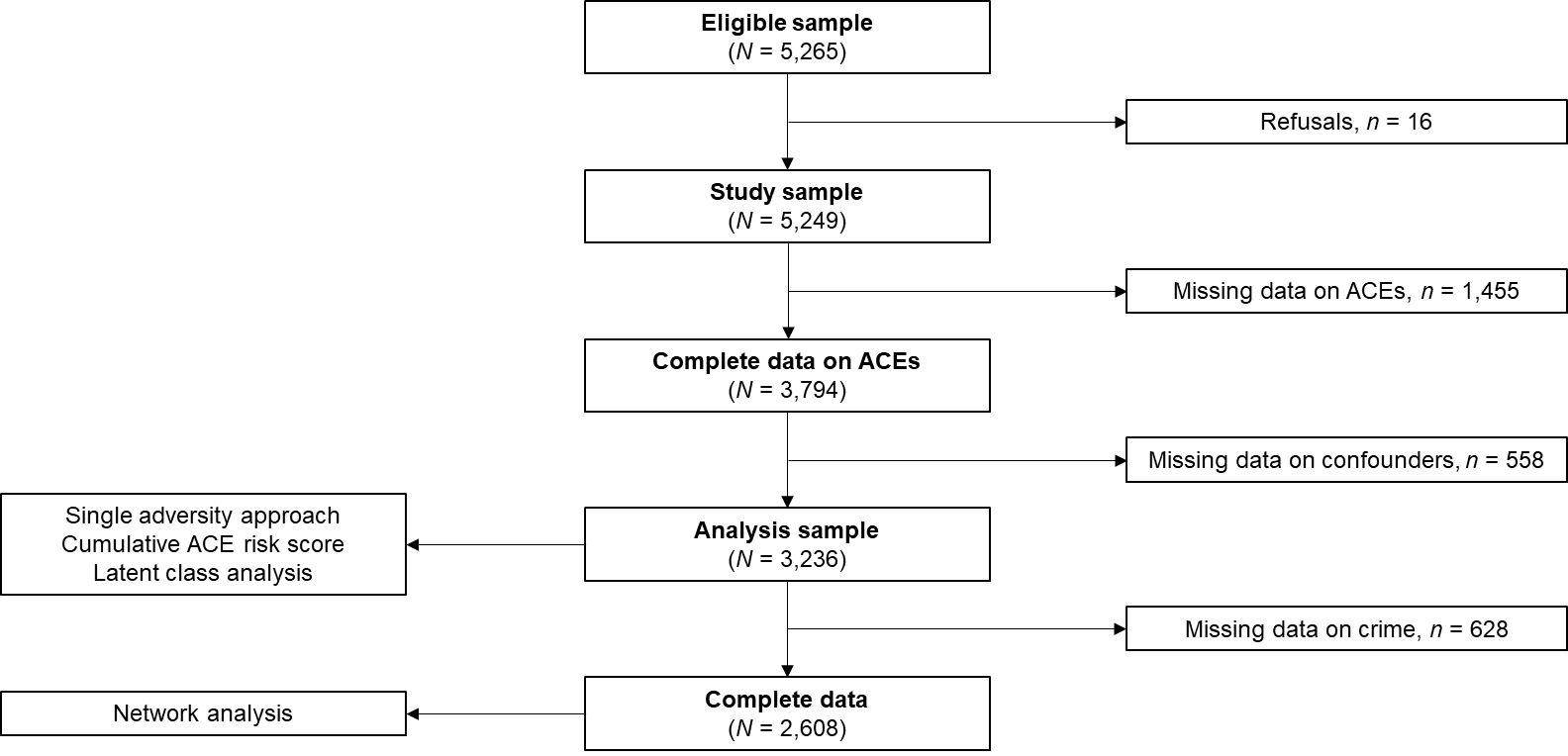


**Note.** ACEs = Adverse childhood experiences.

| **Table S4** Adjusted associations of single adversities and a cumulative adverse childhood experiences risk score with violent and non-violent crime, without using inverse probability weighting | | |
| --- | --- | --- |
|  | **Violent crime**  OR (95% CI) | **Non-violent crime**  OR (95% CI) |
| **Single adversities** |  |  |
| Physical neglect | 1.52 (0.81-2.87) | **2.36 (1.04-5.33)** |
| Physical abuse | **2.46 (1.55-3.91)** | 2.03 (0.99-4.17) |
| Emotional abuse | 1.42 (0.98-2.05) | **1.74 (1.02-2.97)** |
| Sexual abuse | **3.06 (1.14-8.17)** | 2.55 (0.59-11.06) |
| Domestic violence | **1.77 (1.16-2.72)** | **1.98 (1.07-3.67)** |
| Maternal mental illness | **1.70 (1.25-2.31)** | 1.49 (0.93-2.40) |
| Parental divorce | 1.27 (0.94-1.72) | 1.58 (1.00-2.51) |
| Ever separated from parents | 1.13 (0.67-1.91) | 1.00 (0.43-2.35) |
| Parental death | 0.58 (0.27-1.27) | 0.65 (0.20-2.09) |
| Poverty | 1.38 (0.96-1.99) | **1.97 (1.16-3.35)** |
| Discrimination | **1.58 (1.09-2.28)** | 0.87 (0.44-1.70) |
| Neighbourhood fear | 1.37 (0.94-1.98) | 0.88 (0.46-1.68) |
| **Cumulative ACE risk score** |  |  |
| 0 ACE | Ref | Ref |
| 1 ACE | 1.17 (0.76-1.81) | 1.34 (0.66-2.71) |
| 2 ACEs | **1.73 (1.10-2.73)** | 1.71 (0.82-3.59) |
| 3 ACEs | **2.14 (1.29-3.55)** | **2.61 (1.20-5.65)** |
| 4+ ACEs | **2.81 (1.67-4.71)** | **2.82 (1.25-6.36)** |
| ***Note***. Based on available data for adverse childhood experiences and confounders (*N*=3,236). Adjusted for child sex, maternal education, paternal education, and a cumulative score of biological risk factors. Bold values indicate statistically significant results at *p* < .05. | | |

| **Table S5** Unadjusted models across approaches to measuring the association between adverse childhood experiences and violent and non-violent crime | | |
| --- | --- | --- |
| **Analytical approach** | **Violent crime**  OR (95% CI) | **Non-violent crime**  OR (95% CI) |
| **Single adversities** |  |  |
| Physical neglect | 1.78 (0.94-3.36) | **2.48 (1.09-5.64)** |
| Physical abuse | **2.30 (1.45-3.65)** | **2.14 (1.03-4.45)** |
| Emotional abuse | 1.19 (0.83-1.70) | 1.53 (0.90-2.59) |
| Sexual abuse | 1.67 (0.64-4.38) | 2.41 (0.53-10.88) |
| Domestic violence | **1.54 (1.01-2.34)** | 1.78 (0.95-3.31) |
| Maternal mental illness | **1.71 (1.27-2.30)** | 1.58 (0.99-2.54) |
| Parental divorce | 1.21 (0.90-1.63) | **1.74 (1.10-2.76)** |
| Ever separated from parents | 1.17 (0.69-1.98) | 1.27 (0.53-3.02) |
| Parental death | 0.62 (0.28-1.35) | 0.61 (0.19-1.97) |
| Poverty | **1.45 (1.03-2.04)** | **2.08 (1.26-3.43)** |
| Discrimination | **1.49 (1.03-2.16)** | 0.85 (0.42-1.70) |
| Neighbourhood fear | 1.23 (0.85-1.78) | 0.84 (0.43-1.62) |
| **Cumulative ACE risk score** |  |  |
| 0 ACE | Ref | Ref |
| 1 ACE | 1.20 (0.78-1.85) | 1.45 (0.72-2.93) |
| 2 ACEs | **1.64 (1.05-2.56)** | 1.80 (0.86-3.78) |
| 3 ACEs | **1.93 (1.18-3.17)** | **2.51 (1.17-5.37)** |
| 4+ ACEs | **2.25 (1.37-3.70)** | **2.84 (1.28-6.29)** |
| **Latent class analysis** |  |  |
| Low adversities | Ref | Ref |
| Child maltreatment / household challenges | **2.62 (1.40-4.91)** | **3.52 (1.51-8.21)** |
| Household challenges / social risks | **2.29 (1.08-4.86)** | 2.11 (0.67-6.67) |
| ***Note***. Based on available data for adverse childhood experiences and confounders (*N*=3,236) and using inverse probability weighting. ACE = Adverse childhood experience. Bold values indicate statistically significant results at *p* < .05. | | |

**Figure S7** Proportions of individual adversities within each adverse childhood experience score category, including exposure to 1, 2, 3, and 4+ adverse childhood experiences


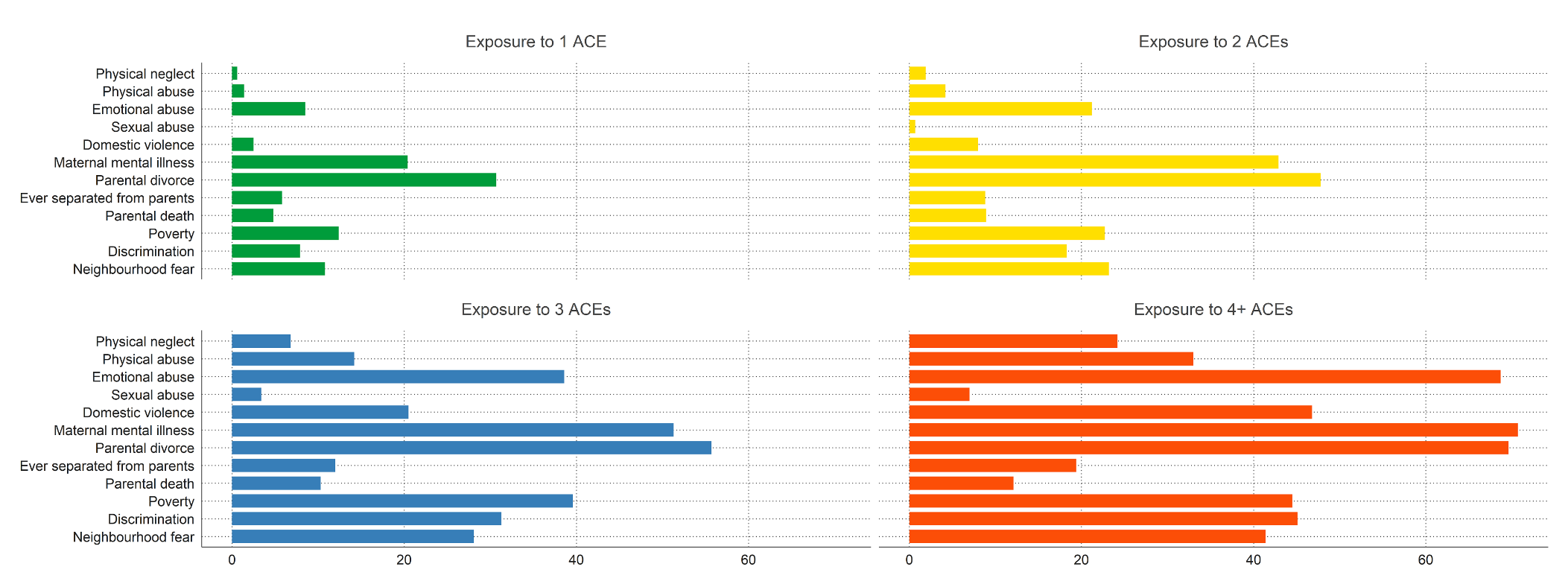


**Note.** ACE =Adverse childhood experience.

**Appendix 4** Information on how latent classes of ACEs were identified

The 3-class model showed the lowest aBIC (27515) but medium entropy (0.57) compared to the 4- and 5-class models, which had slightly higher aBIC values (27518 and 27532, respectively) and a higher entropy (0.70 and 0.70, respectively). While the BLRT showed *p* < 0.001 across all models, favouring higher-class models, the LMR-LRT provided support for the 3-class model, as indicated by non-significant *p*-values for the 4- (0.097) and 5-class models (0.533). The plots of within-class item probabilities between the 3- and 4-class models were almost identical, except for one class in the 3-class model, which was split into two classes in the 4-class model and largely defined by variations in maternal mental illness and parental divorce. Therefore, we selected the 3-class model for subsequent analyses. See Table S5 for more details on model fit statistics, entropy, and class counts and proportions for all class solutions. Considering the relatively poor entropy of the 3-class model, we additionally examined the average probabilities of each class (see Table S6). While classes 1 and 2 showed higher probabilities of 0.81 and 0.84, respectively, class 3 showed a particularly low probability of 0.69, which suggests that 31% of participants assigned to class 3 may not fit that category.

| **Table S6** Model fit statistics, entropy, and class counts and proportions for 1-6 class solutions of adverse childhood experiences | | | | | | |
| --- | --- | --- | --- | --- | --- | --- |
| **Fit statistics** | **1 class** | **2 classes** | **3 classes** | **4 classes** | **5 classes** | **6 classes** |
| Parameters | 12 | 25 | 38 | 51 | 64 | 77 |
| Likelihood | -14235 | -13728 | -13664 | -13634 | -13609 | -13583 |
| aBIC | 28529 | 27580 | **27515** | 27518 | 27532 | 27543 |
| Entropy |  | 0.68 | 0.57 | **0.70** | **0.70** | 0.64 |
| Bivariate fit | 1814 | 274 | 160 | 112 | 97 | 66 |
| LMR-LRT |  | *p* < .001 | *p* = .013 | ***p* = .097** | ***p* = .533** | *p* = .009 |
| BLRT |  | *p* < .001 | *p* < .001 | *p* < .001 | *p* < .001 | *p* < .001 |
| Group size %^a^ |  |  |  |  |  |  |
| C1 | 3236 | 2546 (78.7%) | 2097 (64.8%) | 315 (9.7%) | 158 (4.9%) | 198 (6.1%) |
| C2 |  | 690 (21.3%) | 350 (10.8%) | 311 (9.6%) | 2043 (63.1%) | 791 (24.4%) |
| C3 |  |  | 789 (24.4%) | 2182 (67.4%) | 146 (4.5%) | 379 (11.7%) |
| C4 |  |  |  | 428 (13.2%) | 394 (12.2%) | 228 (7.1%) |
| C5 |  |  |  |  | 496 (15.3%) | 1560 (48.2%) |
| C6 |  |  |  |  |  | 80 (2.5%) |
| ***Note.*** Based on available data for adverse childhood experiences and confounders (*N*=3,236). aBIC = sample-size adjusted Bayesian Information Criterion; BLRT = Bootstrapped Likelihood Ratio Test; C = Class; LMR-LRT = Lo-Mendell-Rubin Likelihood Ratio Test. ^a^ = Final class counts and proportions based on the estimated model. Bold values show key criteria to select the optimal class-model. | | | | | | |

| **Table S7** Average probabilities of each class in the 3-class latent class model on adverse childhood experiences | | | |
| --- | --- | --- | --- |
|  | Class 1 | Class 2 | Class 3 |
| Class 1 | **0.831** | 0.018 | 0.151 |
| Class 2 | 0.031 | **0.838** | 0.131 |
| Class 3 | 0.195 | 0.114 | **0.691** |
| ***Note.*** The diagonal values represent the average posterior probabilities of an assigned class. | | | |

| **Table S8** Adjusted associations between the 3-class model of adverse childhood experiences up to age 15 years and violent and non-violent crime at age 22 years, without using inverse probability weighting | | |
| --- | --- | --- |
|  | **Crime** | |
|  | **Violent**  OR (95% CI) | **Non-violent**  OR (95% CI) |
| Low adversities | Ref | Ref |
| Child maltreatment / household challenges | **3.43 (1.91-6.17)** | **3.97 (1.81-8.68)** |
| Household challenges / social risks | **2.34 (1.19-4.59)** | 2.07 (0.67-6.40) |
| ***Note.*** Based on available data for adverse childhood experiences and confounders (*N*=3,236). Adjusted for child sex, maternal education, paternal education, and a cumulative score of biological risk factors. Bold values indicate statistically significant results at *p* < .05. | | |

| **Table S9** Adjusted associations between the 3-class model of adverse childhood experiences up to age 15 years and violent and non-violent crime at age 22 years, without using inverse probability weighting and based on complete case analysis | | |
| --- | --- | --- |
|  | **Crime** | |
|  | **Violent**  OR (95% CI) | **Non-violent**  OR (95% CI) |
| Low adversities | Ref | Ref |
| Child maltreatment / household challenges | **3.54 (1.94-6.46)** | **3.91 (1.78-8.58)** |
| Household challenges / social risks | **2.31 (1.01-5.28)** | 1.90 (0.56-6.49) |
| ***Note.*** Based on complete data (*N*=2,608). Adjusted for child sex, maternal education, paternal education, and a cumulative score of biological risk factors. Bold values indicate statistically significant results at *p* < .05. | | |

| **Table S10** Multivariable associations of included confounders with adverse childhood experiences and crime outcomes | | | | |
| --- | --- | --- | --- | --- |
|  | **Child sex** (‘male’) | **Maternal education** | **Paternal education** | **Biological risk score** |
|  | OR (95% CI) | OR (95% CI) | OR (95% CI) | OR (95% CI) |
| **Single adversities** |  |  |  |  |
| Physical neglect | **1.44 (1.02-2.03)** | **0.89 (0.83-0.95)** | 0.97 (0.91-1.03) | **1.37 (1.17-1.60)** |
| Physical abuse | **0.70 (0.53-0.93)** | 1.02 (0.97-1.07) | 0.95 (0.91-1.00) | 1.05 (0.92-1.20) |
| Emotional abuse | **0.39 (0.32-0.47)** | **0.95 (0.92-0.98)** | 1.01 (0.97-1.04) | **1.10 (1.01-1.20)** |
| Sexual abuse | **0.25 (0.11-0.50)** | 0.91 (0.81-1.02) | 0.92 (0.82-1.03) | 1.03 (0.78-1.36) |
| Domestic violence | **0.56 (0.44-0.71)** | 1.00 (0.95-1.04) | 1.01 (0.96-1.05) | **1.25 (1.12-1.39)** |
| Maternal mental illness | 1.07 (0.92-1.24) | **0.89 (0.87-0.92)** | 1.00 (0.97-1.03) | **1.24 (1.15-1.33)** |
| Parental divorce | 0.97 (0.84-1.13) | 0.98 (0.95-1.00) | 1.02 (0.99-1.05) | **1.19 (1.11-1.28)** |
| Ever separated from parents | 0.87 (0.67-1.12) | 0.99 (0.94-1.03) | 0.99 (0.95-1.04) | 1.04 (0.92-1.17) |
| Parental death | 1.08 (0.81-1.45) | **0.92 (0.87-0.97)** | 0.96 (0.91-1.01) | 1.04 (0.90-1.19) |
| Poverty | 1.20 (0.99-1.44) | **0.79 (0.76-0.83)** | **0.86 (0.83-0.89)** | **1.16 (1.06-1.27)** |
| Discrimination | 0.88 (0.72-1.06) | **0.96 (0.92-0.99)** | 0.98 (0.94-1.01) | **1.12 (1.02-1.23)** |
| Neighbourhood fear | **0.66 (0.55-0.80)** | 1.00 (0.96-1.03) | 1.01 (0.98-1.05) | **1.10 (1.01-1.21)** |
| **Cumulative ACE risk score** |  |  |  |  |
| 0 | Reference | Reference | Reference | Reference |
| 1 | 1.06 (0.87-1.27) | **0.94 (0.91-0.97)** | 1.00 (0.97-1.03) | **1.12 (1.02-1.23)** |
| 2 | 0.90 (0.73-1.11) | **0.93 (0.90-0.96)** | 0.98 (0.94-1.02) | **1.27 (1.15-1.41)** |
| 3 | **0.75 (0.59-0.96)** | **0.86 (0.82-0.90)** | 0.98 (0.94-1.03) | **1.34 (1.19-1.50)** |
| 4+ | **0.59 (0.46-0.77)** | **0.84 (0.80-0.88)** | 0.96 (0.91-1.00) | **1.53 (1.35-1.72)** |
| **LCA** |  |  |  |  |
| Low adversities | Reference | Reference | Reference | Reference |
| Child maltreatment / household challenges | **0.47 (0.33-0.65)** | **0.86 (0.79-0.94)** | 0.95 (0.89-1.01) | **1.40 (1.22-1.61)** |
| Household challenges / social risks | **1.81 (1.19-2.78)** | **0.72 (0.67-0.77)** | **0.83 (0.77-0.89)** | **1.36 (1.16-1.60)** |
| **Violent crime** | **2.87 (2.13-3.92)** | 0.97 (0.92-1.02) | 1.02 (0.97-1.07) | 1.02 (0.88-1.16) |
| **Non-violent crime** | **2.25 (1.43-3.62)** | 0.95 (0.87-1.03) | 1.01 (0.93-1.09) | 0.97 (0.78-1.19) |
| ***Note.*** Based on available data for adverse childhood experiences and confounders (*N*=3,236) and using inverse probability weighting. Each association between a specific confounder and the exposure(s) and outcome(s) is adjusted for the remaining confounders. Bold values indicate statistically significant results at *p* < .05. | | | | |

**References**

Barreto do Carmo, M. B., dos Santos, L. M., Feitosa, C. A., Fiaccone, R. L., da Silva, N. B., dos Santos, D. N., . . . Amorim, L. D. (2017). Screening for common mental disorders using the SRQ-20 in Brazil: what are the alternative strategies for analysis? *Revista Brasileira de Psiquiatria, 40*(2), 115-122. doi:10.1590/1516-4446-2016-2139

Beusenberg, M., Orley, J., & World Health Organization. (1994). *A user's guide to the self reporting questionnaire (SRQ)*. Retrieved from Geneva, Switzerland: <https://apps.who.int/iris/handle/10665/61113>

Epskamp, S., Borsboom, D., & Fried, E. I. (2018). Estimating psychological networks and their accuracy: a tutorial paper. *Behavior Research Methods, 50*(1), 195-212. doi:10.3758/s13428-017-0862-1

Gomes, A. P., Soares, A. L. G., Kieling, C., Rohde, L. A., & Gonçalves, H. (2019). Mental disorders and suicide risk in emerging adulthood: the 1993 Pelotas birth cohort. *Revista de Saúde Pública, 53*. doi:10.11606/s1518-8787.20190530012356

Mari, J. J., & Williams, P. (1986). A validity study of a psychiatric screening questionnaire (SRQ-20) in primary care in the city of Sao Paulo. *The British Journal of Psychiatry, 148*, 23-26. doi:10.1192/bjp.148.1.23
